# Comment on ‘twenty years of behavioural nutrition – a reflection on the road less travelled’

**DOI:** 10.1186/s12966-025-01821-9

**Published:** 2025-09-30

**Authors:** Marle Alvarenga

**Affiliations:** 1 Post-graduation Program in Public Health Nutrition, School of Public Health, University of São Paulo, São Paulo, Brazil; 2 Instituto Nutrição Comportamental (Behavioral Nutrition Institute), São Paulo, Brazil

**Keywords:** Behavioral nutrition, Eating behavior, Nutrition science, Public health, Dietary interventions

## Abstract

With great interest, I read the article by Lien et al. *“Twenty years of behavioural nutrition – A reflection on the road less travelled”*. This commentary reflects the long-standing absence of a formal definition for “behavioral nutrition” (BN) and its implications for scientific and clinical communities. It highlights the Brazilian experience in conceptualizing BN as a scientifically grounded approach to modifying eating behaviors through a biopsychosociocultural perspective, moving beyond the outdated prescriptive role of the dietitian. The commentary also reviews the historical use of BN in publications, noting inconsistencies, conceptual conflation with “eating behavior,” and limited clarity on what constitutes a BN intervention. A clearer, theory-driven definition and expanded discourse on behavioral approaches and models are essential to advancing the field.

## Main text

With great interest, I read the article by Lien et al. *“Twenty years of behavioural nutrition – A reflection on the road less travelled”* [1]. The publication of a formal definition of behavioral nutrition (BN) in the *International Journal of Behavioral Nutrition and Physical Activity* (IJBNPA) is an important milestone, as the term had previously been used without an explicit definition despite its central role in the journal’s title. As the authors note, BN has often been used interchangeably with diet, nutrition, and eating behaviors, leading to conceptual ambiguity and hindering its establishment as a distinct domain.

In Brazil, the Institute of Behavioral Nutrition (*Instituto de Nutrição Comportamental*) was established in 2014, introducing a clearly articulated BN approach first described in a 2015 book [2]. Before the term was adopted, we reviewed its international use and found only a few instances: (1) the International Society of Behavioral Nutrition and Physical Activity (Netherlands, est. 2002); (2) a U.S.-based eating disorders clinic (behavioralnutrition.org); and (3) the Behavioral Health Nutrition dietetic practice group of the Academy of Nutrition and Dietetics (USA). None provided an explicit definition of a BN.

From its inception, our Institute defined BN as distinct from *eating behavior*, drawing on behavioral psychology and the social psychology of food. We conceptualized BN as a scientifically based approach that applies validated behavioral strategies to facilitate dietary change within a biopsychosociocultural framework. This approach aims to broaden the role of the dietitian, fostering more effective communication and relationships with patients.

BN should be regarded as an approach, not merely an intervention type. This aligns with Lien et al.’s definition, framing BN as the study and promotion of behaviors related to nutrition outcomes. As an approach, a BN provides conceptual scaffolding for interventions rather than a single prescriptive act. For example, the recommendation of a specific food is sometimes labeled a BN intervention, but such prescriptions rarely address the behavioral complexity described by Skinner, which involves environmental contingencies, internal motivations, and learned responses.

Our Institute’s mission is to address *why* and *how* people eat—considering beliefs, thoughts, and feelings—along with *what* and *how much* they eat [2]. Nutritional guidance must integrate behavioral strategies to move beyond rigid prescription models that seldom achieve sustained change. The definition proposed by Lien et al., grounded in Skinnerian principles, aligns closely with our view that BN extends beyond quantitative measures to encompass emotions, cognitions, and psychosocial determinants.

In Brazil, we train nutrition professionals—especially dietitians who still follow prescriptive methods—to integrate behavioral strategies into practice. These include cognitive behavioral therapy, motivational interviewing, dialectical behavior therapy, and nutrition-specific models such as intuitive eating and eating competence, which are systematized in our book [2]. Our framework also incorporates behavioral analysis methods, including chain and functional analyses of eating behaviors, which are consistent with behaviorist traditions.

Although our Institute is primarily educational and clinical, we recognize that research using a BN requires a precise, consensus-based definition. In both psychology and nutrition, *eating behavior*, *eating practices*, and *eating habits* are often conflated, with *eating behavior* sometimes reduced to dietary intake (calories, nutrients, food groups). This narrow view ignores the processes underlying eating, reinforcing the need for a BN to be grounded in behavioral science.

Historically, BN appeared rarely before the IJBNPA’s launch. For example, a 1977 article titled *“Behavioral nutrition: consumption of foods of the future by toddlers”* [3] used BN only in the title, measuring behavior as quantity consumed and tasting—definitions far from a comprehensive behavioral framework. Another example, the NIH program “Biomedical and Behavioral Nutrition Research and Research Training” [4], uses the term without a behavioral science focus. Lien et al. also note a 1979 orthomolecular psychiatry article [5], published in a nonindexed, nonrecommended journal, which conflated a BN with unrelated concepts.

A PubMed search for BN in titles yields mostly IJBNPA papers, with occasional exceptions. Even within the IJBNPA, BN often appears in contexts such as “behavioral nutrition intervention” without a detailed description [6] or as counseling on social and behavioral aspects of eating, guided by models such as the ecological model and social cognitive theory [7]. While these align broadly with BN, heterogeneity in content highlights the need for more explicit parameters.

Furthermore, many interventions labeled as addressing determinants of BN in fact address determinants of *eating behavior*. As the authors rightly state, BN focuses on strategies to change eating behaviors and support adherence to nutritional counseling. In the literature, however, boundaries are blurred, and operationalization does not always reflect the theoretical scope.

A recent IJBNPA commentary on challenges and opportunities for BN researchers [8] did not emphasize enough, in our view, the need for a shared, theory-based definition encompassing behavioral approaches from the social psychology of food. These frameworks have been evaluated for their effectiveness in facilitating change [9, 10] and could strengthen the BN’s conceptual foundations.

## Conclusion

The absence of a clear, universally accepted definition of a BN has resulted in inconsistent application across research and practice. The Brazilian experience demonstrates the value of conceptualizing BN as a comprehensive, theory-driven approach distinct from *eating behavior* and capable of guiding diverse interventions. Advancing the field requires theoretical clarity, the integration of established behavioral models, and consistent operationalization. The IJBNPA is well positioned to lead in defining BN’s scope and methodology, ensuring rigor and coherence in future research.

## Data Availability

No datasets were generated or analysed during the current study.

## Abbreviations

BN: Behavioral Nutrition
IJBNPA: International Journal of Behavioral Nutrition and Physical Activity
